# Impact of treatment policies on patient outcomes and resource utilization in acute cholecystitis in Japanese hospitals

**DOI:** 10.1186/1472-6963-6-40

**Published:** 2006-03-29

**Authors:** Miho Sekimoto, Yuichi Imanaka, Masahiro Hirose, Tatsuro Ishizaki, Genki Murakami, Yushi Fukata, QIP Cholecystectomy Expert Group

**Affiliations:** 1 Department of Healthcare Economics and Quality Management, Kyoto University Graduate School of Medicine, Yoshida Konoe-cho, Sakyo-ku, Kyoto, 606–8501, Japan

## Abstract

**Background:**

Although currently available evidence predominantly recommends early laparoscopic cholecystectomy (LC) for the treatment of acute cholecystitis, this strategy has not been widely adopted in Japan. Herein, we describe a hospital-based study of patients with acute cholecystitis in 9 Japanese teaching hospitals in order to evaluate the impact of different institutional strategies in treating acute cholecystitis on overall patient outcomes and medical resource utilization.

**Methods:**

From an administrative database and chart review, we identified 228 patients diagnosed with acute cholecystitis who underwent cholecystectomy between April 2001 and June 2003. In order to examine the relationship between hospitals' propensity to perform LC and patient outcomes and/or medical resource utilization, we divided the hospitals into three groups according to the observed to expected ratio of performing LC (LC propensity), and compared the postoperative complication rate, length of hospitalization (LOS), and medical charges.

**Results:**

No hospital adopted the policy of early surgery, and the mean overall LOS among the subjects was 30.9 days. The use of laparoscopic surgery varied widely across the hospitals; the adjusted rates of LC to total cholecystectomies ranged from 9.5% to 77%. Although intra-operative complication rate was significantly higher among patients whom LC was initially attempted when compared to those whom OC was initially attempted (9.7% vs. 0%), there was no significant association between LC propensity and postoperative complication rates. Although the postoperative time to oral intake and postoperative LOS was significantly shorter in hospitals with high use of LC, the overall LOS did not differ among hospital groups with different LC propensities. Medical charges were not associated with LC propensity.

**Conclusion:**

Under the prevailing policy of delayed surgery, in terms of the postoperative complication rate and medical resource utilization, our study did not show the superiority of LC in treating acute cholecystitis patients. The timing of surgery and discharge was mainly determined by the institutional policy in Japan, rather than by the clinical course of the patient; however, considering the substantially less postoperative pain and shorter recovery time of LC compared to OC, LC should be actively applied for the treatment of acute cholecystitis. If the policy of early surgery were universally applied, the advantage of LC over OC may be more clearly demonstrated.

## Background

The treatment strategy for acute cholecystitis is now a topic of hot debate. The two main controversies regarding the treatment of acute cholecystitis in patients fit for surgery are the timing of cholecystectomy (either initial conservative treatment followed by delayed cholecystectomy or planned early cholecystectomy), and the selection of the surgical procedure for cholecystectomy (either laparoscopic surgery or laparotomy). The currently available evidence predominantly supports immediate cholecystectomy on the basis that early surgery does not increase the risk of operative morbidity and mortality associated with early cholecystectomy, [1-6] and that such a measure reduces the hospital stay for each patient by up to ten days, in contrast to conservative (late) cholecystectomy. [7,8] Nevertheless, this strategy has not been widely adopted in Japan or in other countries. [9,10]

Another controversy is the selection of laparoscopic surgery in cholecystectomy. Since its introduction, laparoscopic cholecystectomy (LC) has quickly become the most widely used treatment for gallstone disease, because of substantially less postoperative pain and a shorter recovery time compared to open cholecystectomy (OC); [11] however, the indications and contraindications for LC vary widely between providers.[9,10,12-15] In early reports, acute cholecystitis was considered to be an absolute contraindication to this approach. In contrast, subsequent reports showed excellent outcomes for laparoscopic management of acute cholecystitis, and advocated the use of this modality for this population. [16,17] Two randomized clinical trials compared OC and LC for acute cholecystitis and showed mixed results in terms of mortality, morbidity, and postoperative recovery. [18,19] However, little is known about how different strategies for the treatment of acute cholecystitis affect various clinical and patient outcome measures, as well as resource utilization. Therefore, in the present report, we describe the treatment strategies for acute cholecystitis among 9 teaching hospitals in Japan, and investigate the impact of treatment strategies on patient outcomes and hospital revenues.

## Methods

### Subjects and database

The protocol of the present study was approved by the institutional ethics committee of Kyoto University Graduate School of Medicine. The study settings were 9 general hospitals, each of which has more than 350 beds, provides tertiary care, and is certified for residency training. All of the hospitals are members of the Voluntary Hospitals of Japan Quality Indicator Project (VHJ-QIP), which is an initiative for gathering performance data for administrative and quality improvement activities.[10,20,21] These participating hospitals provide the project with administrative data concerning all discharged patients. The administrative database includes patients' demographic data, classification of disease, information about surgeries and complex procedures, length of hospital stay (LOS), and medical claims.

Using this database, we identified 922 consecutive patients who underwent cholecystectomy at one of the participating hospitals between April 2001 and June 2003, and conducted a chart review. First, hospitalizations for cholecystectomy were identified using the appropriate diagnostic codes of the *International Classification of Diseases, Ninth Revision, Clinical Modification (ICD-9CM) *(Open cholecystectomy, 51.21–51.22; Laparoscopic cholecystectomy, 51.23–51.24). From these cases, we selected 254 cases with acute cholecystitis. Acute cholecystitis was defined as "inflammation of the gallbladder" identified by ultrasonography or CT scanning, accompanied by acute attacks of abdominal pain, fever (BT >37.5°C), or laboratory markers of inflammation (white blood cell count = 10,000). [22,23] Cases without gallstones (N = 1), or those complicated with hepatolithiasis (N = 2) were excluded from the study. We also excluded patients who developed acute cholecystitis during hospitalization (N = 3), and those whose clinical or charge data were not perfect (N = 20). Consequently, 228 cases of acute cholecystitis were analyzed.

Two or three independent reviewers collected the following data: complications of choledocholithiasis; comorbidities at admission; execution of percutaneous gallbladder drainage; surgical information including operative procedure (either LC or OC) and operative complications; postoperative clinical course; discharge status; and medical charges. Emergent cases were defined as those complicated by severe jaundice, or with severe local complications such as biliary peritonitis, abscess, empyema, gangrenous or emphysematous cholecystitis. Major intra-operative complications included subcutaneous emphysema, major bleeding (more than 20% of blood loss or bleeding that required transfusion), common bile duct injury, and bowel injury. Major postoperative complications included major postoperative bleeding, bile leakage, bowel obstructions, severe infections such as blood stream infections and pneumonia, acute cholangitis, acute pancreatitis, cardiovascular events, and acute renal failure. The postoperative time to oral intake was calculated by subtracting the date of operation from the date when oral intake was started. When treatment was administered in more than one admission, LOS and medical charges were summed across admissions. Medical charges for ambulatory care were not included in the analyses because data were not available.

### Analysis

Our analysis had 2 primary objectives: (1) to describe hospital variation in the propensity to perform LC; and (2) to examine the relationship between patient outcomes or medical resource utilization and the propensity to perform LC after adjusting for patient risks. For the first objective, our unit of analysis was the individual hospital. For each hospital, we first calculated the observed rate of LC to total cholecystectomies. The use of LC was defined that LC was planned and completed. To account for potential confounding by differences in patient characteristics across hospitals, we used a multivariate logistic regression model to calculate the adjusted rates of performing LC. [24] Variables considered in the model included age, sex, history of upper abdominal surgery, complication of choledocholithiasis, acute pancreatitis or acute cholangitis, comorbidity score (assessed by the Charlson score [25,26]), and acuity of admission (urgent or emergent). Variables significantly associated with the likelihood of undergoing LC in the univariate analysis were included in the final multivariate model. Adjusted rates of LCs to total cholecystectomies were calculated based on observed to expected (O/E) ratios predicted for each hospital by this model.

To assess the relationship between laparoscopy use and patient outcome, the 9 hospitals were first ranked according to their propensity to perform laparoscopic surgery. For each hospital, we assessed 5 primary outcome measures: lengths of stay (total, preoperative and postoperative), major intra-operative complications, major postoperative complications, number of days from surgery to oral intake, and medical charges. We used analysis of variance and multivariate linear regression techniques to calculate adjusted LOS, adjusted postoperative time to oral intake, and adjusted medical charges for each hospital and compared the means across hospitals. We used χ^2 ^tests to assess differences in major postoperative complications, and used multivariate logistic regression techniques to assess differences in the adjusted rate of complications. Independent variables considered in these models and the methods used for selecting variables for the final model are described previously in this section.

## Results

### Variation in patients' backgrounds and the use of LC

In all hospitals, a significant difference was observed in the distributions of age and comorbidity scores of the patients with acute cholecystitis (Table 1 [See Additional file 1]). The proportion of emergent cases did not differ significantly among the hospitals. Either emergent operation or gallbladder drainage was performed in these cases. Only one postoperative death was observed among all subjects, resulting in an overall mortality of 0.3%. Previous studies have defined early operations as those performed within 24 hours to 7 days or those performed on the next operating list; [27] however, no case met the criteria of early surgery except those that underwent emergency operation. The 25th and 50th percentiles for number of days between the onset of symptoms and cholecystectomy were 14 days and 19 days, respectively.

Laparoscopic surgery was planned for 136 (59.7%) patients, and performed on 104 (45.6%) patients, with an overall conversion rate of 23.5%. No hospital in our study performed mini-cholecystecomy. A large variation was observed in the institutional use of LC for patients with acute cholecystitis; the observed rates of LC to total cholecystectomies ranged from 21% to 95%. In multiple logistic regression analysis, the factors significantly associated with the performance of LC included age, history of upper abdominal surgery, and acuity, while sex, comorbidity score, and complication of choledocholithiasis, acute pancreatitis, or acute cholangitis were not significant factors (Table 2 [See Additional file 1]). After adjusting for these factors, LC propensity was still very different among the hospitals; the adjusted rates ranged from 9.5% to 77% (Table 1 [See Additional file 1]). The use of gallbladder drainage also differed significantly among the hospitals; the observed rates of gallbladder drainage ranged from 0% to 54% by hospital.

### Relationships between laparoscopic use and clinical outcomes in patient-level analysis

In patient-level multiple linear regression analysis, the use of LC was significantly associated with 2.4 days shorter time to oral intake (P < 0.001), 7.3 days shorter total LOS (P = 0.001), and 9.3 days shorter postoperative LOS (P < 0.001) after adjusting for patient risk factors such as age, sex, comorbidity, history of upper abdominal surgery, complication of choledocholithiasis, acute pancreatitis, and acute cholangitis, and acuity. In our study, the overall rate of intraoperative complication was significantly higher in patients who requested LC when compared to those whom requested OC (9.7% vs. 0%, P = 0.001). In contrast, the incidence of postoperative complication did not differ when patient age and comorbidity scores were adjusted (P = 0.67).

Gallbladder drainage was more likely to be performed for elderly patients (OR = 1.03; 95%CI, 1.01–1.07, P = 0.05), and was significantly associated with fewer LC (OR = 0.49; 95%CI, 0.26–0.93; P = 0.03), longer preoperative LOS (coefficient, 15.3; 95%CI, 10.7–19.9; P < 0.001), and longer total LOS (coefficient, 13.3; 95%CI, 5.8–20.8; P < 0.001). Surgical fees did not differ widely between LC and OC (US$ 3,713 ± 1,244 for LC and US$ 3,338 ± 1,185 for OC). No significant difference in medical charges was observed between LC and OC after adjustment for patient risks (P = 0.85).

### Relationships between laparoscopic rates and patient outcomes in hospital-level analysis

**Figure 1:**
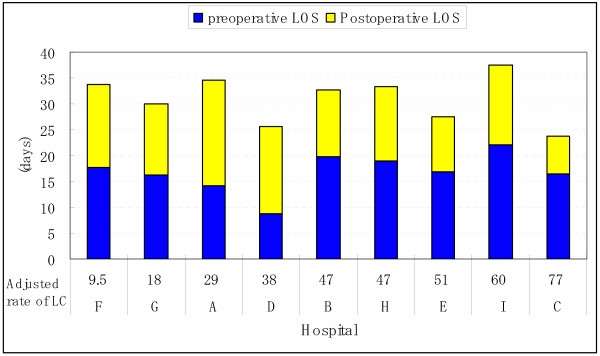
Adjusted rate of LC to total cholecystectomies and adjusted LOS by hospital. Hospitals are sorted by adjusted rate of LC. The blue column represents preoperative LOS, while the yellow column represents postoperative LOS.

Figure 1 [See Figure file] shows the adjusted means for preoperative and postoperative LOS by hospital. In summary, we found no substantial association between hospitals' use of LC and indicators of patient outcome, such as LOS, medical charges, and adjusted rates of postoperative complication. For example, the hospital with the highest use of LC had the shortest average adjusted LOS (both preoperative and total), and the lowest adjusted rate of postoperative complication; however, the hospital with the second highest use had the longest LOS (both postoperative and total).

We then divided the hospitals into three groups according to the O/E ratio of performing LC; the first group included 86 patients of 4 hospitals whose O/E ratios were less than 1.0 (ranging from 0.21–0.83); the second group included 61 patients of 2 hospitals whose O/E ratios were around 1.0 (ranging from 1.02–1.03); and the last group included 81 patients of 3 hospitals whose O/E ratios were above 1.0 (ranging from 1.12–1.69). Table 3 [See Additional file 1] shows the relationship between the propensity of performing LC and patients' characteristics and outcomes.

A significant difference in the distribution of age and comorbidity scores was observed among the three groups. The intra-operative complication rate differed significantly among the groups; the complication rate was lowest among the hospital group with intermediate use of LC, while it was highest in the group with the lowest use of LC. In contrast, the incidence of postoperative comorbidity did not differ. The adjusted mean number of days from surgery to oral intake was significantly different among the three groups; the group with the highest use of LC had about one day shorter time to oral intake compared to the other groups. After adjusting for patients' risk factors, we found a significant difference in preoperative and postoperative LOS among the groups. Preoperative LOS was significantly shorter in the hospital group with the lowest use of LC, while postoperative LOS was significantly short in the group with the highest use of LC. Consequently, there was no difference in overall LOS among the groups. Prevalence of gallbladder drainage did not differ between the groups. However, prevalence of preoperative endoscopic retrograde cholangio-pancreatography (ERCP) differed according to LC propensity; higher use of LC was significantly associated with a higher prevalence of preoperative ERCP. Medical charges were not associated with the propensity for LC.

## Discussion

Comparison of patient outcomes between LC and OC is sometimes difficult because patients' characteristics and risks often differ significantly between the two populations. Currently available evidence has shown mixed results regarding the advantage of LC over OC for patients with acute cholecystitis; thus, interpretation of these results remains controversial. Although the randomized controlled trial conducted by Kiviluoto et al. reported that LC for acute and gangrenous cholecystitis reduces the morbidity rate and sick leave, without increasing the mortality rate, the surgeons who performed LC in this trial were obviously more experienced with the procedure than those who performed OC, which may reduce the validity of this trial. [19] In another randomized trial conducted by Johnson et al., these two approaches were equivalent in terms of low morbidity and rapid postoperative recovery. [18] A study from the United States, which investigated small area variation in the use of LC among elderly patients with AC, has shown that regions with relatively high rates of LC for acute cholecystitis have shorter overall hospital stays (approximately 1 day shorter). [12]

The present study demonstrated a large variation in the treatment strategy for AC, as well as in the LC skills among the hospitals. In addition, these hospitals showed relatively homogeneous performance measures in terms of LOS, and medical charges. Regardless of such a large variation in laparoscopy use, the incidence of major postoperative complications was not associated with LC propensity after adjusting for patients' risk factors. On the other hand, the incidence of intra-operative complications was higher in hospitals with the lowest use of LC. Considering that intra-operative complications occurred only among patients for whom LC was initially attempted, this finding suggests that these complications are more likely to occur during laparoscopic surgery by surgeons with a relatively low LC volume. At the same time, it was suggested that surgeons provide the procedures they think commensurate with their skills and experiences, and such decision-making is appropriate.

The studied hospitals had remarkably long overall hospital stay; the average overall LOS among our study subjects was 30.9 days (range: 21.0 to 38.9 days), while the figures in the reports from other countries range from only 5 to 10 days. [9,12,28] No hospital in our study adopted a policy of early surgery for the treatment of acute cholecystitis during the study period. Reflecting this policy, the studied hospitals had a remarkably long overall preoperative hospital stay; the mean preoperative LOS was 17.1 days, and the institutional means ranged from 8.0 to 21.3 days; however, if the policy of early surgery were to be universally applied, we consider that the overall LOS would be reduced by more than 10 days. Unfortunately, in Japan, early surgery could impose a heavy burden on hospitals that suffer from a lack of human resources.

In our data, the impact of LC use on the reduction of LOS was small. Although postoperative LOS was significantly shorter in hospitals with a high use of LC when compared to those with low and intermediate use of LC, longer preoperative LOS in this group negated the advantage of LC for reducing postoperative hospitalization. There are some controversies regarding the impact of laparoscopic surgery on the reduction of morbidity and hospital stays. For example, several studies have suggested that mini-cholecystectomy is a safe, inexpensive day surgery method requiring minimal time off work after surgery, which is comparable to LC, and questioned the application of this technique for all cases. [29,30] However, these studies did not limit subjects to patients with acute cholecystitis, and therefore, the results may not be generalizable to the discussion regarding the treatment of acute cholecystitis.

Kehlet et al. reviewed articles investigating "fast-track surgery", which include modifying perioperative care, such as pain control, the introduction ofinnovative techniques that reduce the perioperative stress response, and more frequently use of minimal invasive surgical access. [31] They critically reviewed randomized trials comparing "open" and "laparoscopic" procedures, and indicated that traditional care regimens have been infrequently revised in the open treatment group. Therefore, they argue that the studies in which LOS and convalescence were utilized as endpoints may merely reflect traditions of perioperative care associated with open procedures rather than differences between open and closed surgical techniques.

The present study also revealed that the timing of surgery and discharge are primarily determined by institutional policy, rather than by the clinical course of the patient. For example, the timing of surgery was not related to either the use of LC or the use of gallbladder drainage. Moreover, the hospitals' postoperative complication rates were not associated with the mean postoperative LOS of the hospital, although they were significantly associated with the mean postoperative time to oral intake. Such findings suggest that the timing of discharge is determined by quite different criteria among the providers, and that the length of hospitalization is not necessarily an indicator of clinical performance in Japan.

The prevailing policy of delayed cholecystectomy with an accompanying long hospitalization for patients with acute cholecystitis may be attributable to the unique medical system of Japan. In Japan, hospitals provide not only acute care but also intermediate care and long-term care. [32] Traditionally, surgical patients in Japan have stayed in the hospital until they have completed convalescence, at which time they can resume their normal life. Although this tradition is now changing due to pressure from the government and a shift in the medical payment system, many healthcare providers continue to follow their traditional care regimen. Moreover, longer hospital stays do not necessarily bring economic disadvantages to hospitals. Since medical cost are usually reimbursed to hospitals on a fee-for-service basis, a longer hospital stay yields economic incentives for the hospital. Therefore, the utilization of new technologies does not necessarily bring economic benefits. For example, laparoscopic procedures are often more time-consuming than traditional procedures, at least during the 'learning curve', and they are also usually more expensive compared to open procedures, mainly due to higher operating room costs for laparoscopic surgeries. [33]

## Conclusion

Although our analysis documents a wide variation in LC rates, it does not by itself answer the question, "Which rate is right?" The institutional postoperative complication rates, means of overall LOS and medical charges in our study suggest that these two techniques are comparable in terms of medical resource utilization. Contrary to the results from clinical trials, in the real world, where surgeons' skills and patients' characteristics are much more diverse than in experimental settings, the advantages of LC have no direct bearing on patient outcomes. At the same time, however, there is no doubt that LC is still superior to OC in terms of less postoperative suffering and less pain for the patient. [34-36] Considering that the incidence of postoperative complication and medical resource utilization is comparable between OC and LC, LC may be a superior strategy for the treatment of acute cholecystitis. Unfortunately, the current healthcare payment system in Japan is based on a fee-for-service model and provides no economic incentive for healthcare organizations to reduce the length of hospitalization or to reduce unnecessary medication and laboratory testing. Reform of the medical system, including the separation of acute care from intermediate care and nursing care, as well as economic support for efficient medical resource utilization, is necessary for facilitating the standardization of treatment for acute cholecystitis based on scientific evidence.

## List of abbreviations used

LOS: length of stay

LC: laparoscopic cholecystectomy

OC: open cholecystectomy

LC propensity: propensity to perform laparoscopic cholecystectomy

O/E ratio: observed to expected ratio

## Competing interests

The author(s) declare that they have no competing interests.

## Authors' contributions

MS and YI conceived the study; MS, GM, and YF independently collected clinical information, while MS MH and IT analyzed the data. All authors contributed to the analysis independently, and interpretation of the results was discussed until final agreement.

## Pre-publication history

The pre-publication history for this paper can be accessed here:



## Supplementary Material

Additional File 1 1124466370860534_Tables.doc (Microsoft Word document; Tables 1 through 3) Click here for file
