# Supplementary material for: Impact of treatment policies on patient outcomes and resource utilization in acute cholecystitis in Japanese hospitals
Source: BMC Health Serv Res. 2006 Mar 29;6:40. doi: 10.1186/1472-6963-6-40 (PMC1488841; doi:10.1186/1472-6963-6-40)
Supplement: Additional File 1 — 1124466370860534_Tables.doc (Microsoft Word document; Tables 1 through 3) [file 1472-6963-6-40-S1.doc]

Table 1. Patient characteristics by hospital

| Hospital | A | B | C | D | E | F | G | H | I | Total | P value |
| --- | --- | --- | --- | --- | --- | --- | --- | --- | --- | --- | --- |
| No of cases | 14 | 29 | 30 | 22 | 30 | 22 | 28 | 32 | 21 | 228 |  |
| Age | 67.7±11.2 | 66.2±12.6 | 57.2±15.8 | 60.6±14.0 | 67.9±11.3 | 67.1±13.9 | 71.0±12.9 | 67.1±14.1 | 60.6±11.6 | 65.1±13.7 | 0.003 |
| Male, % | 50 | 66 | 53 | 73 | 63 | 55 | 61 | 53 | 62 | 60 | 0.85 |
| Gallbladder drainage, % | 29 | 38 | 37 | - | 20 | 45 | 54 | 47 | 19 | 33 | 0.001 |
| Previous upper abdominal surgery, % | 29 | 17 | 3 | 5 | 3 | 9 | 14 | 3 | 10 | 9 | 0.09 |
| Complication of Choldicholithiasis | 29 | 17 | 10 | 18 | 17 | 23 | 25 | 25 | 14 | 19 | 0.82 |
| Acuity (Emergent), % | 36 | 45 | 40 | 41 | 30 | 45 | 54 | 47 | 24 | 41 | 0.55 |
| Charlson score | 0.14±0.53 | 0.03±0.19 | 0.37±0.61 | 0.05±0.21 | 0.23±0.43 | 0.05±0.21 | 0.39±0.74 | 0.13±0.34 | 0.19±0.40 | 0.18±0.46 | 0.02 |
| Rate of LC to cholecystectomies, % |  |  |  |  |  |  |  |  |  |  |  |
| Observed | 27 | 61 | 90 | 50 | 81 | 22 | 29 | 67 | 95 | 62 | <0.001 |
| Adjusted | 29 | 47 | 77 | 38 | 51 | 9 | 18 | 47 | 60 | 46 |  |
| Conversion rate, % | 0 | 37 | 0 | 18 | 29 | 60 | 50 | 21 | 25 | 24 | 0.017 |
| Gallbladder drainage, % | 29 | 38 | 37 | 0 | 20 | 45 | 54 | 47 | 19 | 33 | 0.001 |
| Postoperative morbidity, % (adjusted) | 21 | 7 | 7 | 18 | 10 | 9 | 11 | 22 | 10 | 12 | 0.563 |
| Mean length of day from surgery to oral intake, d (adjusted) | 3.7 | 2.4 | 2.3 | 3.1 | 1.9 | 2.7 | 3.5 | 4.5 | 2.1 | 2.9 | 0.01 |
| Mean length of stay, d (adjusted) |  |  |  |  |  |  |  |  |  |  |  |
| total | 36.4 | 31.9 | 24.6 | 25.9 | 27.5 | 34.5 | 30.6 | 33.0 | 36.5 | 30.9 | 0.01 |
| preoperative | 14.1 | 19.9 | 16.4 | 8.7 | 16.9 | 17.8 | 16.2 | 19.0 | 22.1 | 17.1 | 0.0001 |
| Postoperative | 20.4 | 12.9 | 7.5 | 17.0 | 10.7 | 16.0 | 13.8 | 14.3 | 15.4 | 13.8 | 0.15 |
| Mean medical charge (adjusted, US$ 1,000) | 11.5 | 10.4 | 10.4 | 9.3 | 10.5 | 10.8 | 10.9 | 11.9 | 12.3 | 10.9 | 0.04 |

Table 2. Logistic regression analysis regarding factors associated with application of LC

| Factor | Odds Ratio | (95% Confidence Interval) | P |
| --- | --- | --- | --- |
| Age | 0.97 | (0.95-0.99) | 0.01 |
| History of upper abdominal surgery | 0.05 | (0.01-0.39) | 0.005 |
| Emergent case | 0.27 | (0.15-0.50) | <0.001 |
| Sex | 1.47 | (0.80-2.7) | 0.22 |
| Chralson score | 0.90 | (0.48-1.7) | 0.74 |
| Choledocholithiasis | 0.73 | (0.31-1.7) | 0.47 |
| Acute pancreatitis | 1.50 | (0.35-6.4) | 0.58 |
| Acute cholangitis | 2.87 | (0.62-13.3) | 0.18 |

Table 3. Patient characteristic and outcomes by hospital’s propensity of performing LC.

|  | Propensity to perform LC | | | P |
| --- | --- | --- | --- | --- |
|  | High | Middle | Low |  |
| No of hospitals/cases | 3 / 81 | 2 / 61 | 4 / 86 |  |
| Age | 62.0±13.9 | 66.7±13.3 | 66.9±13.5 | 0.04 |
| Male, % | 59 | 59 | 61 | 0.98 |
| Previous upper abdominal surgery, % | 4.9 | 10 | 12.8 | 0.21 |
| Complication of Choledocholithiasis | 13.6 | 21.3 | 23.3 | 0.26 |
| Acuity (Emergent), % | 32 | 46 | 45 | 0.14 |
| Charlson score | 0.27±0.50 | 0.08±0.28 | 0.17±0.51 | 0.05 |
| Rate of LC to cholecystectomies, % |  |  |  |  |
| Observed | 73 | 44 | 21 | <0.001 |
| Adjusted | 63 | 47 | 23 | <0.001 |
| Conversion rate, % | 17 | 29 | 33 | 0.15 |
| Gallbladder drainage, %  Observed | 34 | 37 | 28 | 0.11 |
| Preoperative ERCP, %  Observed | 49 | 25 | 19 | <0.001 |
| Intra-operative complication, %  Observed | 4.9 | 0 | 9.3 | 0.05 |
| Postoperative morbidity, %  Adjusted | 10 | 13 | 13 | 0.82 |
| Mean length of stay, d (adjusted) |  |  |  |  |
| total | 28.8 | 32.5 | 31.4 | 0.21 |
| preoperative | 18.0 | 19.4 | 14.5 | 0.01 |
| Postoperative | 10.7 | 13.7 | 16.2 | 0.0019 |
| Mean length of day from surgery to oral intake | 2.1 | 3.5 | 3.2 | 0.005 |
| Mean medical charge (adjusted, 1000 fee-point) | 129 | 132 | 125 | 0.58 |
